# The myofibroblast in wound healing and fibrosis: answered and unanswered questions

**DOI:** 10.12688/f1000research.8190.1

**Published:** 2016-04-26

**Authors:** Marie-Luce Bochaton-Piallat, Giulio Gabbiani, Boris Hinz

**Affiliations:** 1 Department of Pathology and Immunology, Faculty of Medicine, University of Geneva, Geneva, Switzerland; 2 Laboratory of Tissue Repair and Regeneration, Matrix Dynamics Group, Faculty of Dentistry, University of Toronto, Toronto, Canada

**Keywords:** Myofibroblast, mechanotransduction, myofibroblast generation, myofibroblast contraction, hypertrophic scars, organ fibroses

## Abstract

The discovery of the myofibroblast has allowed definition of the cell responsible for wound contraction and for the development of fibrotic changes. This review summarizes the main features of the myofibroblast and the mechanisms of myofibroblast generation. Myofibroblasts originate from a variety of cells according to the organ and the type of lesion. The mechanisms of myofibroblast contraction, which appear clearly different to those of smooth muscle cell contraction, are described. Finally, we summarize the possible strategies in order to reduce myofibroblast activities and thus influence several pathologies, such as hypertrophic scars and organ fibrosis.

## Introduction

Wound healing has interested the medical praxis since the beginning of human history, but for many centuries the effort of physicians has concentrated more on empirical therapeutic strategies rather than on the understanding of its biological mechanisms. During the last few centuries, however, a gradual progress has been achieved in defining and understanding several physiological aspects of wound healing. In particular, the formation and evolution of granulation tissue has been described in the second half of the 18th century, mainly thanks to the British surgeon John Hunter, and in the last century it has been shown that wound contraction is due to an active contraction of granulation tissue, mainly thanks to the work of the French surgeon Alexis Carrel ^1^. The discovery of the myofibroblast more than forty years ago allowed the identification of the cell responsible for this phenomenon ^2^. This coincided with the early establishment of the cytoskeleton concept ^3^. The myofibroblast was then considered to be a contractile non-muscle cell ^4^. Since the first description, our knowledge of myofibroblast structure and activity has progressed enormously. The purpose of this article is to briefly summarize the biological features of the myofibroblast and to discuss some of the promising strategies to suppress this cell’s activity in order to achieve the possibility of influencing important pathological situations, such as fibrotic lesions, that presently cannot be cured successfully.

## Evolution of the myofibroblast concept

Initially, the myofibroblast was described by means of electron microscopy revealing the presence of prominent cytoplasmic microfilament bundles and peripheral focal adhesions in the fibroblastic cells of granulation tissue ^2^. Electron microscopy further showed the existence of gap junctions connecting myofibroblasts, thus reinforcing the suggestion of similarity between myofibroblasts and smooth muscle (SM) cells ^5^. The production of a specific antibody against α-SM actin, the actin isoform typical of vascular SM cells, allowed the demonstration that myofibroblasts express α-SM actin and are hence equipped with a typical SM protein ^6^.

In the early phases of granulation tissue formation after the production of a wound, local fibroblasts begin moving from the unaffected dermis and subcutaneous tissue toward the wound center and acquire bundles of microfilaments, similar to *in vitro* stress fibers, containing only β- and γ-cytoplasmic actins; these cells have been named proto-myofibroblasts and evolve generally into α-SM actin containing differentiated myofibroblasts that are responsible for wound contraction ^7^. When the wound closes, myofibroblasts disappear through apoptosis ^8^, and a scar persists in the affected area. When myofibroblasts persist in a closed wound, they indicate the development of a hypertrophic scar, an important pathological evolution of wound healing, particularly frequent after burn injury ^7, 9^. Myofibroblasts are also present in all fibrotic diseases, such as scleroderma, as well as liver, kidney, and lung fibrosis and are prominent in heart failure and repair after myocardial infarction. Finally, myofibroblasts are the main components of the stromal reaction to several epithelial tumors ^7, 10^. It should be noted that both proto-myofibroblasts and differentiated myofibroblasts can be found in normal tissues, for example in lung alveolar septa and at the periphery of intestinal crypts, respectively ^7^, where they probably exert physiological mechanical functions.

Much work has been performed in order to find specific markers of the myofibroblastic phenotype. As stated above, α-SM actin discriminates myofibroblasts from fibroblasts and has become the most used marker for this cell. Several other markers have been proposed, but no specific marker has been identified until now. However, several SM cell markers are not expressed in myofibroblasts, such as SM myosin heavy chains, h-caldesmon, and smoothelin ^11^; this underlines the functional differences between the two cells, as we shall discuss below.

## Mechanisms of myofibroblast formation and evolution

**Figure 1.**
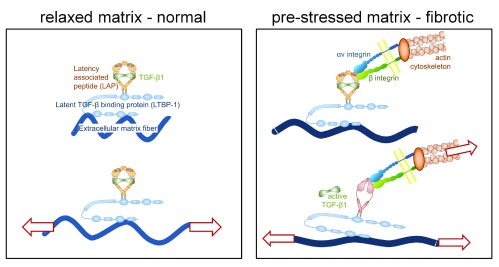
Mechanical activation of TGF-β1 In normal connective tissue, loosely arranged collagen protects resident fibroblasts and latent transforming growth factor (TGF)-β1 complexes from being strained with the extracellular matrix (ECM). Fibroblasts in normal tissue do not express or present the integrin receptors that bind and activate latent TGF-β1. During tissue repair and in organ fibrosis, activated myofibroblasts express αv integrins that connect the contractile actin/myosin cytoskeleton to latent TGF-β1. The accumulation of collagen and its excessive remodeling (crosslinking) by these myofibroblasts result in denser and straighter ECM fibers, which leads to overall higher tissue stiffness. Because ECM fibers are straighter, even smaller strains applied to the fibrotic ECM externally, or by residing myofibroblasts, will be sufficient for the release of active TGF-β1 (modified from Hinz B and Suki B [2016] *Does breathing amplify fibrosis?* Editorial on 21).

After a wounding insult, blood extravasation and clot formation occur followed by an inflammatory phase that allows an accumulation of blood-borne cells, liberating many cytokines and growth factors essential for the onset of the following phase of granulation tissue formation ^12^. In early granulation tissue, motile proto-myofibroblasts appear and start to synthesize extracellular matrix (ECM) components, such as collagen type I and III ^7^. Another relevant new component of ECM is cellular fibronectin, which contains the alternatively spliced segments EDA (or EIIIA) and EDB (or EIIIB) and is present in connective tissue during development but reappears in pathological situations such as granulation tissue and fibrotic lesions ^13, 14^. EDA fibronectin has been shown to be essential for the differentiation of myofibroblasts ^15^. The early transformation of fibroblasts into proto-myofibroblasts appears to depend on the mechanical changes taking place in the wound compared to the normal skin, in particular increased stiffness ^7, 16^; moreover, platelet-derived growth factor has been shown to stimulate proto-myofibroblast motility ^17^. The development of α-SM actin synthesizing differentiated myofibroblasts is essentially due to the action of transforming growth factor (TGF)-β1 in the presence of EDA fibronectin ^15, 18^. TGF-β1 is present in the ECM as a large latent complex including latency-associated peptide and latent TGF-β1-binding protein ^9^. It can be liberated by proteolytic enzymes as well as by integrin-dependent mechanically induced mechanisms ^9^. The force exerted by stress fibers through transmembrane integrins is enough to free TGF-β1 from the large latent complex, and the strained ECM is capable of maintaining a feedback mechanism, assuring a persistent fibrotic activity by the myofibroblast ^19, 20^; moreover, straining and/or stiffening of the ECM can increase the availability of TGF-β1 ^21, 22^ ( Figure 1). Straining and stiffening are consequences of fibroblast and myofibroblast remodeling activities. Matrix stiffening is additionally promoted by fibroblast and inflammatory cell-derived collagen crosslinking enzymes including lysyl oxidases and lysyl oxidase-like enzymes, as reviewed in 20, 23. The incorporation of α-SM actin into stress fibers has been shown to significantly increase the contractile activity of fibroblasts ^24^; the force generated by myofibroblast stress fibers is transmitted to the ECM through focal adhesions that contain specialized transmembrane integrins ^25^. As stated above, myofibroblasts disappear when a wound closes, mainly through apoptosis ^8^. The mechanisms of apoptosis induction, or conversely of myofibroblast persistence, in hypertrophic scars are not clarified; however, the importance of a focal adhesion complex component, Hic-5, a paxillin homologue, in maintaining the myofibroblast phenotype has been demonstrated ^26^. Moreover, myofibroblasts can disappear by means of accelerated senescence ^27^ and even, at least in some instances, revert to the normal phenotype ^28^.

## Mechanism of myofibroblast contraction and mechanotransduction

The initial studies suggesting a similarity between myofibroblast and SM cell contractile activities ^4^ were gradually reconsidered in light of the consideration of the different functional activities of the two cells: SM cell contraction is rapid and short in duration, whereas myofibroblast contraction is rather long lasting and results in a permanent tissue retraction, probably stabilized by ECM deposition ^7^. Evidence has gradually accumulated suggesting that, in addition to the classical calcium-calmodulin-myosin light chain kinase-dependent SM cell contraction mechanism ^11^, myofibroblast contractile activity can be regulated by the activation of the Rho/ROCK/myosin light chain phosphatase pathway ^7, 29– 31^. This long-duration type of contraction underlines an essential difference between the SM cell and the myofibroblast and could explain the characteristic tissue remodeling activity of this cell.

The forces generated by the contractile activity of myofibroblasts are transmitted to the surrounding ECM through specialized focal adhesions containing transmembrane integrins. As a result, strained and more compacted ECM develops. Interestingly, the mechanical conditions generated by the myofibroblast feedback leads to their sustained pro-fibrotic activity ^10, 19^. More recently, it has been shown that megakaryoblastic leukemia factor 1 (MKL1), also named myocardin-related transcription factor (MRTF), is crucial for myofibroblast differentiation and mechanotransduction. In various myofibroblast precursor cells, it links mechanical stress to the transcriptional activity of muscle-cell genes via the polymerization state of actin ^32– 36^. Inhibition of MRTF reduces experimentally induced skin fibrosis in rodents ^37^, as well as differentiation of human colonic myofibroblasts ^38^. Similarly, YAP/TAZ transcription factors, known to mediate mechano-responses ^39^, positively regulate myofibroblast activation ^40– 44^.

## Myofibroblast origin

One of the intriguing features of the myofibroblast is that it can derive from a large variety of cell types. As stated above, mesenchymal cells with myofibroblastic features are present in normal tissues, including the uterine submucosa, follicles of lymph nodes and spleen, intestinal villous cores and crypts, theca externa of the ovary, periodontal ligament, adrenal capsule, lung septa, and bone marrow stroma ^7^. It appears more and more evident that the term fibroblast comprises a heterogeneous cell population ^10, 45^, thus it is possible that only some specialized fibroblastic cells generate myofibroblasts in normal and pathological situations, as recently supported by studies on skin and heart fibrosis ^46– 48^. During pathological situations, local fibroblasts allegedly represent the major source of myofibroblastic cells ^10^; however, in particular cases, other local cells become the main precursors, such as SM cells in coronary atheromatous plaque ^49^, keratocytes in the eye ^50, 51^, perisinusoidal cells in the liver ^52^, and pericytes in many organs ^53– 55^. In addition, myofibroblasts may develop through the process of epithelial-mesenchymal transition ^56, 57^ or endothelial-mesenchymal transition ^58^. Finally, myofibroblasts may derive from circulating bone marrow-derived specialized inflammatory cells called fibrocytes and participate in fibrotic lesions in several organs ^59, 60^. Circulating and/or resident mesenchymal stromal/stem cells (MSCs) are prominent precursors of myofibroblasts in a variety of organs and injury situations ^61, 62^. Because delivery of MSCs is an attractive approach to regenerate organs that are beyond repair within the body’s own capacity ^63, 64^, understanding MSC-to-myofibroblast activation (fibrogenesis) will be of particular importance for the success of MSC therapies ^40, 65^.

There is considerable variability and dispute in the literature concerning the proportions of different precursor cells contributing to the myofibroblast pool. However, different research groups seem to generally agree that myofibroblast sources can differ between different individuals, organs, animals, or particular injury models. For example, in the corneal fibrosis model, 30 to 70% of myofibroblasts are derived from bone marrow-derived precursors depending on the type of wound and the individual that is wounded (reviewed in 51). Thus, using drugs that modulate myofibroblast activation from specific precursor cells can be an effective strategy to inhibit fibrosis in an organ-specific manner.

## Perspectives

As we have seen, the myofibroblast represents an eclectic cell whose major function appears to be the remodeling of connective tissue. If we consider the variety of its possible origins, the myofibroblast could be defined as a phenotypic variant of many cell types, developing upon the appearance of appropriate stimuli. Myofibroblast activity can be physiological, e.g., regulation of ventilation/perfusion ratio in pulmonary alveoli, and useful for wound healing but noxious in many pathological situations, e.g., fibrotic lesions ^7^.

Despite many attempts and despite the clinical importance of fibrotic lesions ^9, 12^, there is not at present any clinically accepted pharmacological tool capable of influencing myofibroblast activity and thus the evolution of these diseases. We shall discuss some strategies that could possibly lead to the development of efficient tools. Despite the heterogeneity of origin, all differentiated myofibroblasts perform the same functions, i.e. tissue remodeling and synthesis of ECM. Hence, the processes regulating these functions appear to represent promising targets of therapeutic strategies. TGF-β1 would appear as an ideal target in order to control myofibroblast activity. Unfortunately, until now no relevant results have been obtained by using direct inhibitors; however, several pathways of TGF-β1 action remain to be explored and a number of clinical trials that target TGF-β1 are pending ^66^. EDA fibronectin is necessary for myofibroblast differentiation ^15^ and its absence results in wound healing or pulmonary fibrosis reduction ^67^, suggesting that EDA fibronectin could be addressed as a therapeutic target. The observation that α-SM actin is essential for the remodeling activity of the myofibroblast and the finding that its N-terminal peptide Ac-EEED is essential for α-SM actin incorporation into stress fibers ^68^ have suggested that this peptide could represent a tool for decreasing myofibroblast activity. This possibility has been demonstrated *in vitro* and in rat wound healing ^69^, suggesting that this peptide or, possibly more efficiently, a mimetic compound could be used therapeutically. The recent observation that tropomyosin 1.6/7 isoforms play an essential role in the stable incorporation of α-SM actin into fibroblast stress fibers ^70^ points to a new target for the reduction of α-SM actin expression in myofibroblasts with the consequent reduction of their remodeling activity ^71^. Another way to regulate myofibroblast remodeling activity could be the control of the Rho/ROCK/myosin light chain phosphatase pathway. In this respect, it has been shown that the ROCK inhibitor Y-27632 decreases granulation tissue contraction ^31^. Closely related to reducing cell contraction is the idea of blocking myofibroblast adhesion to the ECM via integrins. This will have two potential beneficial outcomes: reduced force transmission to the ECM and, provided the correct integrins are targeted, reduction of TGF-β1 activation ^72, 73^.

Further work is needed in order to develop an efficient therapeutic approach to excessive wound healing and fibrotic diseases. Importantly, myofibroblast research will need to cross organ boundaries to exploit the full potential of drugs that are effective in one system but not studied in others. For instance, mitomycin C was shown to block fibrosis development after eye surgery ^74^, but its action in other organs is unknown. We feel confident that during the next few years the above-discussed strategies will allow the discovery of new, efficient tools to control these devastating diseases.
